# Purified IgG from aquaporin-4 neuromyelitis optica spectrum disorder patients alters blood-brain barrier permeability

**DOI:** 10.1371/journal.pone.0238301

**Published:** 2020-09-03

**Authors:** Alvaro Cobo-Calvo, Anne Ruiz, Chloé Richard, Sandrine Blondel, Sylvie Cavagna, Nathalie Strazielle, Jean-François Ghersi-Egea, Pascale Giraudon, Romain Marignier

**Affiliations:** 1 Service de Neurologie, Sclérose en Plaques, Pathologies de la Myéline et Neuroinflammation and Centre de Référence Pour les Maladies Inflammatoires Rares du Cerveau et de la Moelle (MIRCEM)–Hôpital Neurologique Pierre Wertheimer Hospices Civils de Lyon, Lyon, France; 2 Centre de Recherche en Neurosciences de Lyon, U1028 INSERM-CNRS UMR5292-UCBL, Bron, France; 3 Centre d’Esclerosi Múltiple de Catalunya (Cemcat), Department of Neurology/Neuroimmunology, Hospital Universitari Vall d’Hebron, Universitat Autònoma de Barcelona, Barcelona, Spain; 4 BIP Facility, CRNL, Lyon, France; Hungarian Academy of Sciences, HUNGARY

## Abstract

**Background:**

Neuromyelitis optica spectrum disorders (NMOSD) is a primary astrocytopathy driven by antibodies directed against the aquaporin-4 water channel located at the end-feet of the astrocyte. Although blood-brain barrier (BBB) breakdown is considered one of the key steps for the development and lesion formation, little is known about the molecular mechanisms involved. The aim of the study was to evaluate the effect of human immunoglobulins from NMOSD patients (NMO-IgG) on BBB properties.

**Methods:**

Freshly isolated brain microvessels (IBMs) from rat brains were used as a study model. At first, analysis of the secretome profile from IBMs exposed to purified NMO-IgG, to healthy donor IgG (Control-IgG), or non-treated, was performed. Second, tight junction (TJ) proteins expression in fresh IBMs and primary cultures of brain microvascular endothelial cells (BMEC) was analysed by Western blotting (Wb) after exposition to NMO-IgG and Control-IgG. Finally, functional BBB properties were investigated evaluating the presence of rat-IgG in tissue lysate from brain using Wb in the rat-model, and the passage of NMO-IgG and sucrose in a bicameral model.

**Results:**

We found that NMO-IgG induces functional and morphological BBB changes, including: 1) increase of pro-inflammatory cytokines production (CXCL-10 [IP-10], IL-6, IL-1RA, IL-1β and CXCL-3) in IBMs when exposed to NMO-IgG; 2) decrease of Claudin-5 levels by 25.6% after treatment of fresh IBMs by NMO-IgG compared to Control-IgG (p = 0.002), and similarly, decrease of Claudin-5 by at least 20% when BMEC were cultured with NMO-IgG from five different patients; 3) a higher level of rat-IgG accumulated in periventricular regions of NMO-rats compared to Control-rats and an increase in the permeability of BBB after NMO-IgG treatment in the bicameral model.

**Conclusion:**

Human NMO-IgG induces both structural and functional alterations of BBB properties, suggesting a direct role of NMO-IgG on modulation of BBB permeability in NMOSD.

## Introduction

Neuromyelitis optica spectrum disorders (NMOSD) is a severe autoimmune disease of the central nervous system (CNS) that mainly affects the optic nerve and the spinal cord. The discovery of a serum antibody, termed aquaporin-4 antibody, directed against the aquaporin4 (AQP4) channel expressed at the CNS interfaces, has enhanced the understanding of NMOSD which is now considered an autonomous entity with distinctive pathophysiology, different from multiple sclerosis [1].

Contrary to the extended evidence concerning AQP4-IgG involvement in NMOSD tissue lesion formation, the mechanisms for antibody penetration into the CNS, still awaits elucidation. Over the last few years, four main hypotheses have been proposed to explain the passage of immunoglobulins (NMO-IgG) of NMOSD patients, from either serum or cerebrospinal fluid (CSF) into CNS. First, NMO-IgG has been observed to access the brain from the blood (blood-CNS barrier) through fenestrated endothelial cells in circumventricular organs such as the area postrema where AQP4 protein is highly expressed [2,3]. Although, NMO-IgG deposition was initially thought to be restricted to the area postrema [2], a recent study, using a monoclonal murine AQP4-antibody with high antigen affinity, found a wide diffusion in the CNS [4]. Second, *in vitro* models have shown that either the NMO-IgG itself [5,6], or other components from the serum of NMOSD patients (such as matrix-metalloproteinase 2/9 protein, antibodies against brain endothelial cells, or glucose-regulated protein-78) may alter the blood brain barrier (BBB) at the glio-vascular unit (blood-CNS barrier) [7–9]. Third, NMO-IgG might gain access to the CNS via the CSF using the paravascular pathway (CSF-CNS barrier) [10] by which the end-feet of astrocytes would be directly exposed to CSF circulating NMO-IgG, allowing their entrance into the CNS [11]. In fact, higher levels of NMO-IgG have been found in the CSF of NMOSD patients at relapse compared to remission phases [12]. Finally, extravasation of the antibody through meningeal and small parenchymal vessels has been recently proposed as new route for antibody entry into the CNS [4].

In the field of NMOSD, the impact of NMO-IgG on BBB has mainly been evaluated using *in vitro* assays [5–8]. However, the use of *ex vivo* models could provide a more precise representation of the antibody behaviour when reaching the BBB, and a better understanding of the underlying pathophysiology.

Herein, an *ex vivo* approach consisting of fresh brain microvessels isolated from rat brain to model the blood-CNS barrier was used for the first time to assess the impact of NMO-IgG on the BBB. A first analysis was performed to determine whether NMO-IgG from AQP4-positive NMOSD patients may orchestrate BBB alteration by inducing a specific cytokine secretome profile in isolated brain microvessels (IBMs). Then, using both IBMs and an *in vivo* animal model simulating CSF-CNS barrier, the structural modifications of the BBB induced by NMO-IgG were investigated. Finally, to evaluate whether such structural modifications are sufficient to induce a breakdown of the barrier an *in vitro* bicameral model was set-up to simulate the blood-CNS barrier.

## Materials and methods

### Patients, IgG-patient purification, and NMO-IgG selection

De-identified serum specimens were obtained from six different relapsing NMOSD patients that were included in the French cohort of NMOSD (NOMADMUS) and stored at NeuroBioTec (Biological Resource Center of the Hospices Civils de Lyon, France). All patients were tested positive for AQP4-antibodies detected by cell-based assay [13] and all patients fulfilled the 2015 criteria for NMOSD [14]. Serum provided by the French blood service (Etablissement Français du Sang) from healthy blood donors were used as controls. Both NMO-IgG (called NMO-IgG_1-6_) and healthy donors-IgG (called Control-IgG) were purified from the NMOSD and healthy donor serums, respectively, using chromatography over Protein–A Sepharose as previously described [15]. IgGs were then used at a final concentration of 200 μg/mL.

### Intraventricular brain NMO-IgG infusion, and ethical procedures

Animal surgery was performed as previously described [15]. Briefly, rats were anesthetized and mounted in a stereotaxic frame. Two hundred μl of purified IgG (NMO-IgG_1_, 2 mg/ml) or NaCl (Control-IgG) were infused into the CSF during seven days at a rate of 1 μL/hour using a sterile mini-osmotic pump implanted subcutaneously (Osmotic pump ALZET, Charles Rivers, France). After seven days, rats were anesthetised and received an intracardiac perfusion of 100 mL phosphate-buffered saline (PBS; 0.1 M, pH 7.4). Then, rats were sacrificed using pentobarbital, brain was then removed and frozen in isopentane at -30°C, and further stored at -80°C.

Brain tissue was embedded in Tissue-Tek OCT and subsequently cut on a cryomicrotome [15]. Brain slices were used for immunohistochemical (IHC) study of tight junction proteins.

NMO-rats were defined as those receiving NMO-IgG and control-rats as those receiving NaCl. Animal care and procedures were carried out in accordance with the European Directive 2010/63/UE and followed the Animal Research Reporting of *In Vivo* Experiments (ARRIVE) guidelines. The study was approved by the local Lyon 1 University Animal Care Committee (B2012-80 project).

### Isolation of brain microvessels

Isolation of brain microvessels was performed from two adult rat brains per experiment according to the procedure described [16]. Adult rats were anesthetized with isoflurane and decapitated. Brains were excised and placed at 4°C in Krebs-Ringer buffer (in Mm: 135NaCl, 4 KCl, 2.2 CaCl_2_, 1.2 MgCl_2_,6 NaHCO_3_, 10 Hepes, 5 Glucose monohydrate, pH 7.4). Meninges and choroid plexuses were dissected. Using oxygen-saturated buffers, cortices were homogenised in a Dounce-type glass-glass homogeniser. The preparation was further homogenised in 5 vol/g tissue of 1% BSA-supplemented Kreps Ringer buffer (AKRB), and then filtered through a 500μm mesh sieve (Netwell, Corning, Corning, NY). The filtrate was diluted with 1% AKRB (1:1) and homogenised again. Then, the homogenate was centrifuged for 10 minutes, the pellet was suspended in Krebs-Ringer buffer containing 17.5% 70 kD-dextran, and further centrifuged for 27 minutes. Myelin was retained at the surface of the gradient and the resulting pellet was suspended in 1% AKRB. Pellet was consecutively filtered through 200 and 74-μm mesh sieves to eliminate larger vessels. The preparation was centrifuged for 15 minutes. Finally, the pellet was suspended and filtered on a 40μm mesh sieve (BD Bioscience, Erembodegem, Belgium). All procedures were performed at 4°C.

The fresh IBMs retained were recovered in AKRB 0.1% and 2 μl were used to observe their morphology under the microscope. Immunofluorescence analysis showed the presence of Claudin-5, Occludin and ZO-1 in fresh IBMs preparation (S1 Fig). Fresh IBM suspended in 0.1% AKRB were centrifuged for 15 min at 800 rpm and immediately suspended in 2ml of *Dulbecco's Modified Eagle's (DMEM) + BSA0*.*1%*. To determine the *ex vivo* effect of NMO-IgG on tight junction proteins (Claudin-5, Occludin, and ZO-1), fresh IBMs together with *DMEM/BSA 0*.*1% were distributed in 6 different 24-well plates (*330 μl *in each well) and further treated by adding either* 330 μl of DMEM/BSA *0*.*1% (called non-treated IBMs)*, *Control-IgG (2 mg/ml)*, *or NMO-IgG*_1_ *(2 mg/ml)*. *In each well the medium volume was completed to 1 ml with* DMEM and the IBMs were incubated for 9 hours and 20 hours at 37°C prior to collection by centrifugation for cytokine/chemokine analysis and Wb, respectively.

### Brain microvascular endothelial cell and astrocyte cultures

Brain microvascular endothelial cells (BMEC) were isolated as previously described [17]. Briefly, microvessels were isolated from 3 rat brains following mechanical and enzymatic digestion of brain tissue, and plated in a T75 flask coated with collagen and fibronectin (microvessels from 1.5 cortex per T75 flask). BMEC were grown and purified with decreasing concentrations of puromycin for 5 days, followed by 3 days of culture in an insulin, transferrin and sodium selenite-containing medium. The cells were cultured on plastic for BMEC experiments or harvested for seeding on filters to generate *in vitro* BBB models, as follows. After dissociation during 30 seconds with trypsin 0.05%-EDTA 0.02% solution at 37°C per T75 flask, the detached BMEC (approximately 3*10^6^cells/T75) were dissociated by pipetting up and down. Re-suspended BMEC were then added to the upper compartment of a pre-coated well filters filled with endothelial cell media (ECM) at high density (160*10^3^cells/filter of 1.1cm^2^). Endothelial cells expressed tight junction proteins within 3 days [17]. BMEC were then exposed in the luminal part to DMEM/BSA0.1% (termed non-treated BMEC), Control-IgG or NMO-IgG_1-6_ for 20 hours at the concentration of 350 μg/ml.

As previously described, [15] primary glial cultures were isolated from 1-day-old rat pups (n = 24) and dissociated cells were further diluted in DMEM to a density of 2.10^5^ cells. Cells were seeded in 6-well plates and incubated at 37°C in a moist 5% CO_2_, 95% air atmosphere. In order to obtain pure astrocyte cultures and eliminate microglia and oligodendrocytes, cells were treated with cytosine arabinoside (AraC, 25nM, Sigma-Aldrich).

### Permeability analyses: Bicameral model

For antibody permeability analyses, BMEC were exposed to three different NMO-IgG 200 μg/mL in the upper compartment of the culture system. The upper, luminal compartment of the model represents the blood, while the lower, abluminal compartment represents the brain interstitial fluid. After 3 hours the acceptor compartment was collected and frozen for later NMO-IgG analysis by Enzyme-linked immunosorbent assays (ELISA). The amount was divided by the initial concentration in the donor compartment, time of incubation and surface area of the cell monolayer, to generate a permeability coefficient expressed as μl/hour/cm^2^.

To test the integrity of the barrier, [14C] sucrose was added in the upper compartment at the end of the incubation with IgG, and sucrose transfer was measured over a 1-hour period. The radioactivity was measured over time in the lower compartment using a Perkin Elmer TRICARB, 4910TR liquid scintillation analyser, Singapore. Permeability coefficient was calculated as described [18]. Briefly, the amount of sucrose that reached the acceptor compartment was determined for each time point, and divided by the concentration of the molecule in the solution added in the donor compartment. Clearance values were plotted over time, and permeability coefficients, expressed as μl/min/cm^2^, were calculated as the slope of the clearance curve divided by the cell-covered surface area available for transfer.

### Enzyme-linked immunosorbent assays

The level of human IgG was quantified in the acceptor compartment by ELISA set according to the manufacturer's protocols (Human IgG ELISA Quantitation Set, Bethyl Laboratories, Inc, Montgomery, Alabama, USA). Samples were diluted at 1:15. The optical density was measured at 450 nm with *λ* correction of 570 nm using a Spark spectrophotometric microplate reader (Tecan Trading AG, Switzerland). Based on the optical density value of each sample, the sample concentration was calculated in ng/mL and used to calculate clearance.

### Cytokine analysis

Evaluation of cytokines and chemokines from fresh IBMs supernatants were assayed in two replicates by using Proteome Profiler Array kit (rat cytokine array panel A, R&D Systems, Minneapolis, MN, USA) according to the manufacturer’s instruction. Chemiluminescence signal intensity was measured by ImageJ software.

### Immunocytochemistry procedure

Cytospined IBMs were fixed in 4% paraformaldehyde for 10 minutes, then washed in PBS, and blocked for 1 hour in blocking solution (10% Normal Goat serum, *PBS* 1X, BSA 1%, Triton 0.3%). Then, the material was incubated overnight at 4°C with primary antibodies in blocking solution and further washed in PBS. Slides were labelled at room temperature with specific fluorescent secondary antibodies in blocking solution for 30 minutes, washed in PBS and incubated with 4',6-diamidino-2-phenylindole (DAPI). Slides were mounted in buffered glycerol and stored at 4°C. Axio Imager Z1 Apotome technology (Zeiss, Oberkochen, Germany) was used to image the slides.

### Immunoblotting

Fresh IBMs, BMEC and brain tissues collected on sliced rat brain at the limit of the lateral ventricular wall (micro-punches on parenchymal tissue) were dissociated by ultrasound fragmentation in homogenisation buffer and phosphatases inhibitors, and then protein content was counted, as previously described [15]. Protein samples were separated by sodium dodecyl sulphate-polyacrylamide *gel electrophoresis* (SDS-PAGE gels) and transferred to nitrocellulose membranes. Beta-actin was used as an internal standard. The membranes were treated with blocking buffer for 1 hour and incubated with the specified primary antibodies at 4°C overnight. Membranes were exposed to an anti-IgG antibody for 1-h at room temperature and revealed using chemiluminescence, as described [15]. Quantification of the band intensity was obtained using ImageJ software.

### Immunohistochemical reagents

The following IgGs were used for tissue sample immunodetection and lysates. The primary antibodies were; Claudin-5 (mouse monoclonal, 35–2500; Invitrogen), Occludin (rabbit polyclonal, 71–1500; Invitrogen), ZO-1 (rabbit polyclonal, 61–7300; Invitrogen), PDGF Receptor-β (rabbit monoclonal, 28E1; Cell Signalling Technology), PECAM-1 (purified rat, MEC 13.3; BD Pharmigen), GFAP (rabbit Z0334, Dako Denmark A/S), AQP4 (rabbit polyclonal AB2218; Merck© KGaA), beta-Actin (mouse monoclonal, A1978; Sigma-Aldrich®). The secondary antibodies were: Peroxidase-AffiniPure F(ab)2 fragment donkey anti-mouse IgG (Heavy[H]+light [L] chains) (715-036-151, Jackson Immunoresearch), Alexa Fluor®488 goat anti-mouse IgG, A11029; Alexa Fluor®555 goat anti-mouse IgG, A21424; Alexa Fluor®488 goat anti-rabbit IgG, A11034; and Alexa Fluor®455 goat anti-rabbit IgG, A21429 from Molecular Probes Inc.

### Statistical analysis

All Wb analyses were performed by investigators blinded for treatment conditions. Data are presented as median (interquartile range [IQR]) and percentages. For assessment of the expression of tight junction protein by Wb among different conditions, a non-parametric Kruskal-Wallis test was used to evaluate differences among the non-treated BMEC, Control-IgG and NMO-IgG_1._ Then, a U-Mann-Whitney test was used to compare Control-IgG or NMO-IgG to non-treated. A Spearman test was performed to evaluate correlations between AQP4 and Claudin-5 expression on astrocytes and BMEC, respectively, after exposing to NMO-IgG_1-6_. All statistical analyses were performed using Prism 5.0 GraphPad Inc software.

## Results

### 1. Selection of NMO-IgG

**Fig 1:**
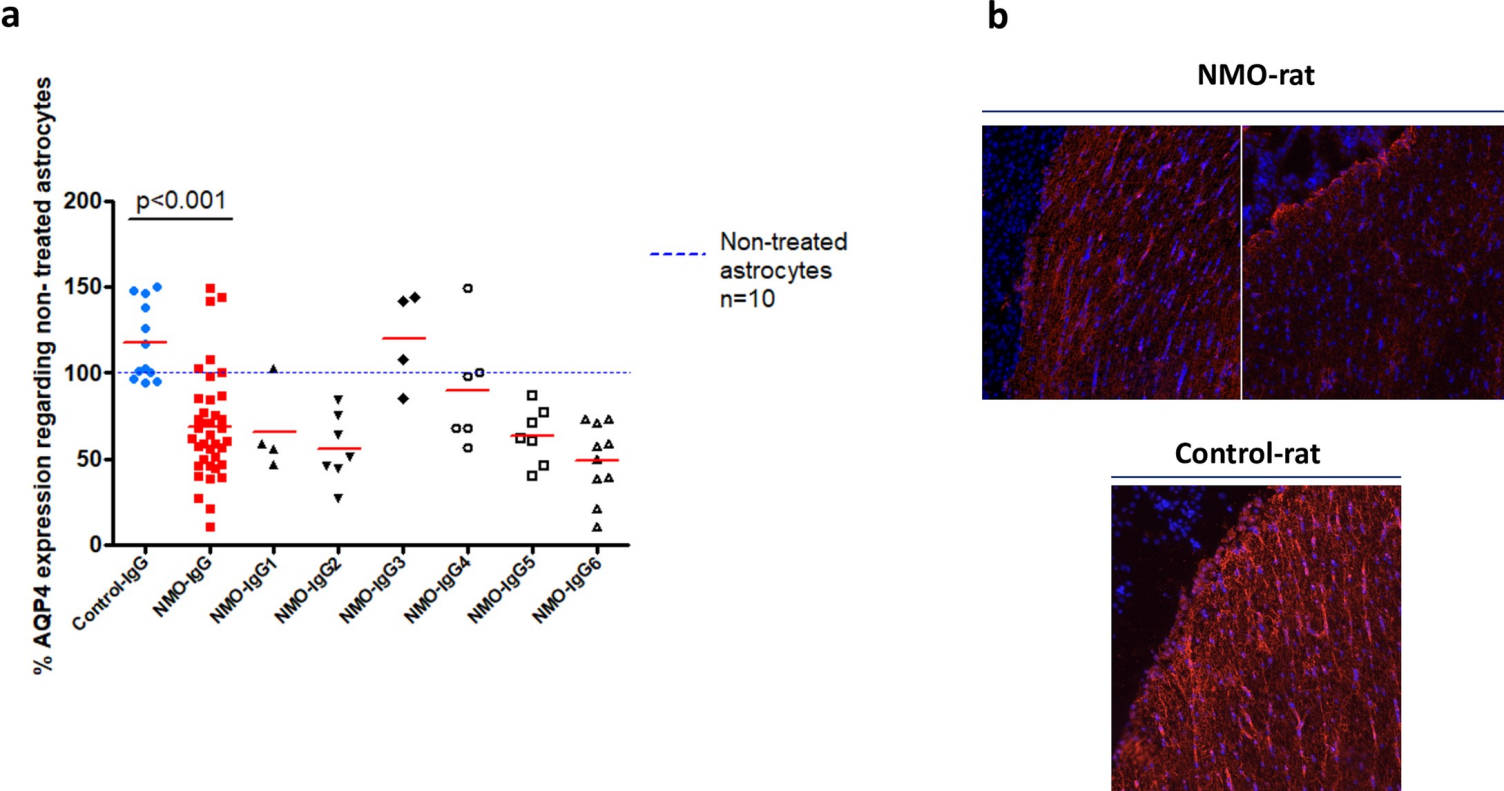
Aquaporin-4 expression in astrocyte culture and rat brain tissue. (a) Expression of AQP4 in astrocytes after 24 hours of treatment with NMO-IgG_1-6_ (n = 39) compared to treatment with Control-IgG (n = 12). Dash blue line represents non-treated astrocytes (Wb). (b) Example of AQP4 expression in the periventricular regions of rat brains after being treated with NMO-IgG (NMO-rat) compared to Control-IgG (Control-rat) (immunohistochemistry).

NMO-IgG from the different patients were first tested on cultured astrocytes in order to select NMO-IgG for the experiments on fresh IBMs and for the animal model (NMO-rat). After 24 hours of treatment with NMO-IgG_1-6_ (number of experiments = 39) there was a significant decrease in cultured astrocyte AQP4 expression (mean 114.9±24.3) when compared to treatment with Control-IgG (n = 12, mean 70.3±17.9, p<0.001), as measured by western blot (Wb) (Fig 1A). NMO-IgG also induced a decrease in AQP4 expression in the periventricular regions of rat brains when compared to Control-IgG (Fig 1B).

The effects of NMO-IgG_1-6_ and Control-IgG were evaluated in duplicate or triplicate. NMO-IgG_1,2,6_ were the most pathogenic, inducing a higher decrease in AQP4 expression than NMO-IgG_3,4,5_. Among the most pathogenic NMO-IgG, NMO-IgG_1_ was randomly selected to carry out experiments.

### 2. Purified IgG from NMOSD patients induces an inflammatory secretome profile in brain microvessels

**Fig 2:**
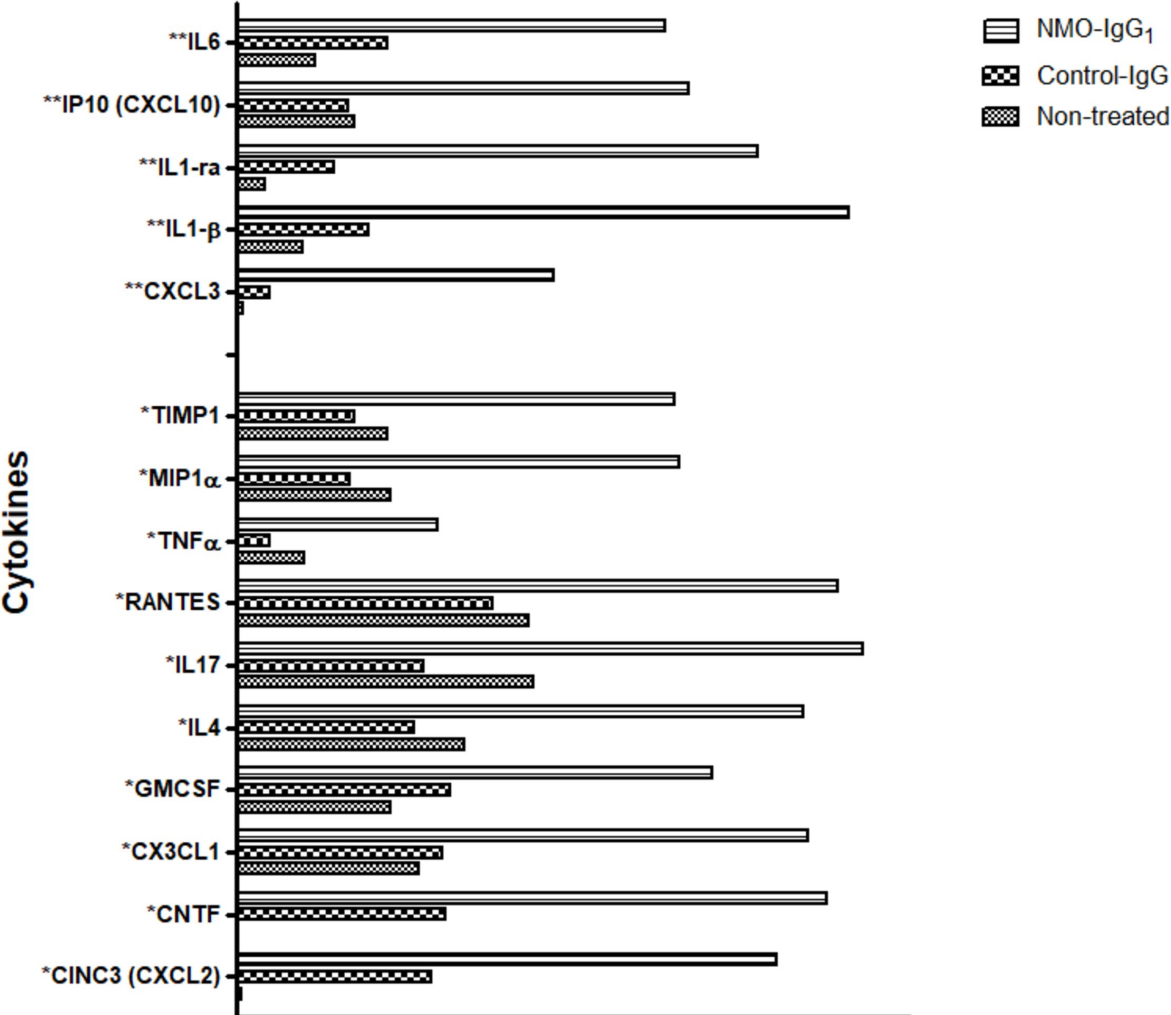
Cytokine/chemokine levels in the supernatant of IBMs after 9 hours of treatment with NMO-IgG_1_, Control-IgG and those of non-treated microvessels. *2 and **3-fold increase in fluorescence signal intensity in NMO-IgG_1_ vs. Control-IgG.or non- treated microvessels. IgG were added at the final concentration of 200 μg/mL. Protein levels were assessed using membrane arrays and the measured densitometric signals are expressed as arbitrary units.

Because soluble factors secreted by the BBB cells may auto-modulate BBB function in the presence of NMO-IgG, the cytokine secretome profile of one batch of fresh IBMs in contact with NMO-IgG_1_ was evaluated. Cytokine/chemokine protein array was performed on IBMs supernatants treated with NMO-IgG_1_ and Control-IgG. Mean signal intensity of IL-6, IL-1RA, IL-1β, CXCL10 (IP-10) and CXCL3 showed at least a three-fold increase in IBMs exposed to NMO-IgG_1_ compared to control conditions (non-treated fresh IBMs or Control-IgG). For the ten following cytokines/chemokines, TNFα, RANTES, IL-17, IL-4, GM-CSF, CNTF, MIP1α, CX3CL1, CXCL1/2 and TIMP1, the signal intensity increased at least two-fold (Fig 2).

Globally, exposition to purified IgG from AQP4-antibody positive NMOSD patients resulted in the production of an inflammatory secretome profile from brain microvessels.

### 3. Purified NMO-IgG induce Claudin-5 loss in brain microvessels

**Fig 3:**
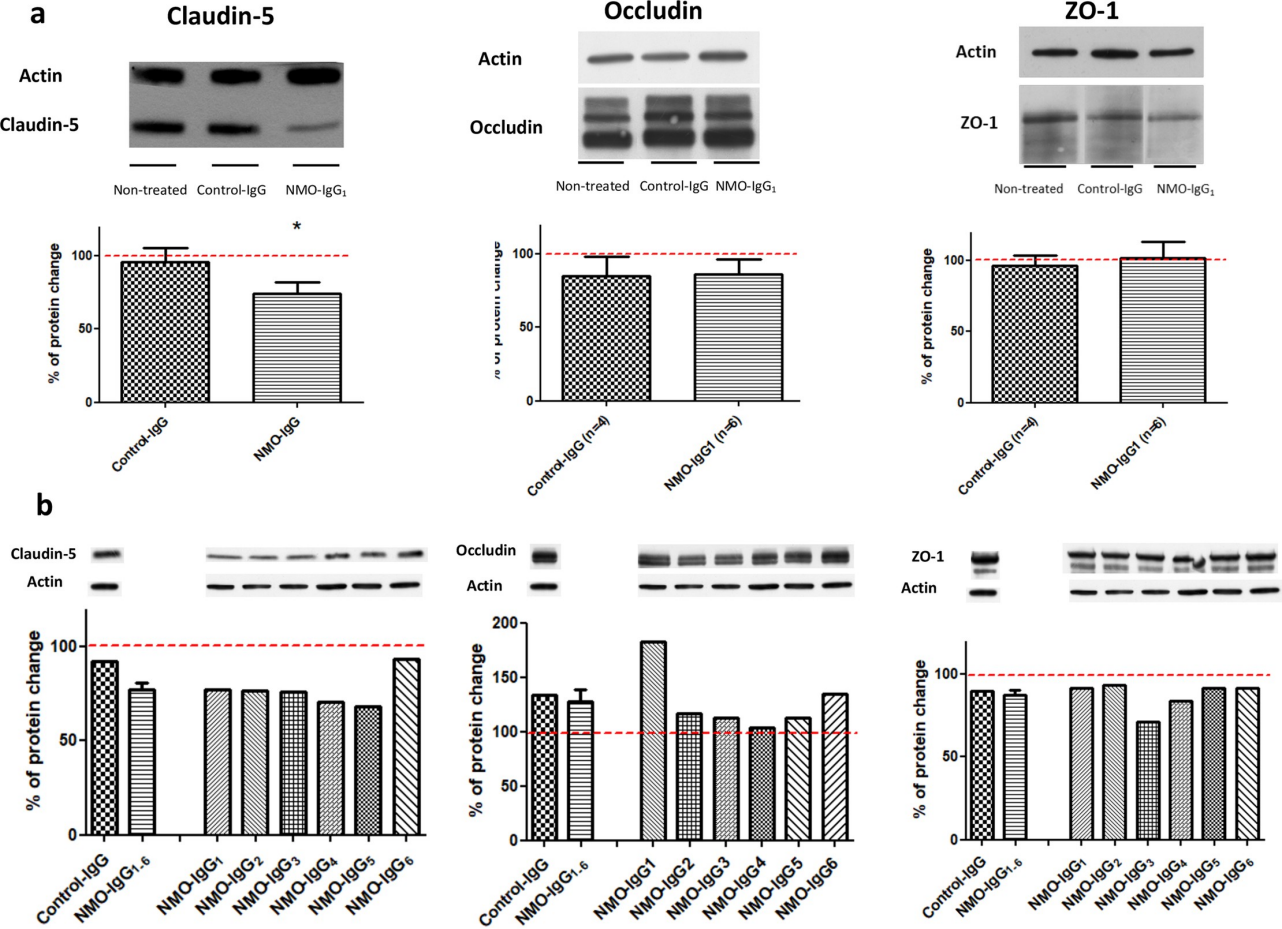
Tight junction protein expression detected using Wb in IBMs and endothelial cell cultures treated with NMO-IgG. (a) Expression of tight junction proteins in fresh non-treated IBMs (n = 6) and in IBMs treated for 20 hours with NMO-IgG1 (n = 6) and Control-IgG (n = 4). The Kruskal- Wallis probe showed significance differences in the expression of Claudin-5 among non-treated BMEC, Control-IgG and NMO-IgG1 treated BMECs, p = 0.040. Assessment of Occludin and ZO-1 expression in these three conditions showed not significant difference. A significant decrease in Claudin-5 expression in the presence of NMO-IgG1 compared to non-treated IBMs was observed (NMO-IgG1, 25.61%; p = 0.002). No difference was observed between Control-IgG and non-treated IBMs [U-Mann-Whitney test]). *p-value <0.05. (b). Expression of tight junction proteins in BMECs after 20 hours of treatment with Control-IgG or NMO-IgG. Immunoblotting showed a Claudin-5 decrease by at least 20% in five out of the six NMO-IgG conditions (NMO-IgG1-5). Non-treated freshly IBMs (reference category) are depicted as a red line both in panel (a) and (b).

Assuming that the inflammatory environment induced by NMO-IgG has the capacity to alter the BBB, Claudin-5, Occludin, and ZO-1 protein expression analysis was performed in non-treated IBMs (n = 6) and in fresh IBMs treated with NMO-IgG_1_ (n = 6), and Control-IgG (n = 4), respectively, for 20 hours (Fig 3A). Immunoblotting showed a significant 25.6% decrease in Claudin-5 expression in the presence of NMO-IgG_1_ compared to *non-treated IBMs* (p = 0.002), while it was not observed in the Control-IgG treated group (3.9%; non-significant) [U-Mann-Whitney test]). There were no significant modifications regarding Occludin and ZO-1 expression.

These findings were confirmed in primary BMEC cultures following 20-hour treatment with the six NMO-IgG_1-6_ (Fig 3B). Immunoblotting showed that Claudin-5 expression decreased by at least 20% in five out of the six NMO-IgG conditions (NMO-IgG_1-5_).

Immunohistochemical analysis of Claudin-5 in IBMs treated with NMO-IgG_1_ showed that Claudin-5 was still present within an apparent normal junctional localization in the microvascular network (data not shown).

Overall, these results indicate that purified IgG from AQP4-antibody positive NMOSD patients modify the expression of the tight junction protein Claudin-5.

**Fig 4:**
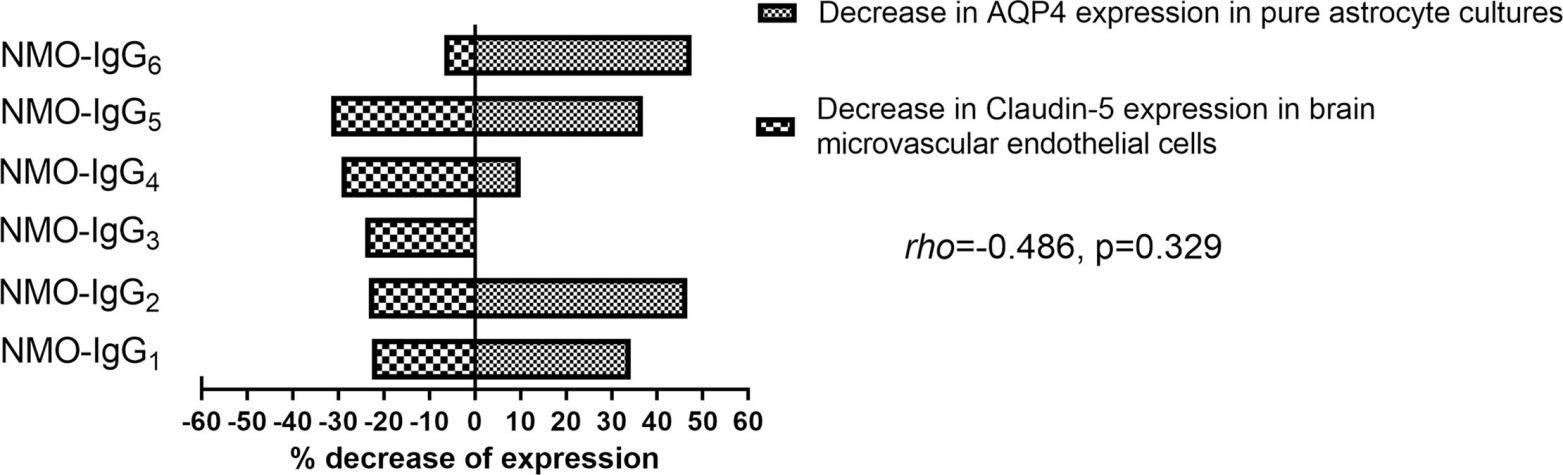
AQP4 and Claudin-5 expression tested in astrocytes and BMECs, respectively. A Spearman correlation was performed between AQP4 and Claudin-5 expression on astrocytes and BMECs after exposing them to NMO-IgG_1-6_, respectively.

When comparing the effects of each NMO-IgG_1-6_ on AQP4 expression in astrocytes and on Claudin-5 expression on BMEC, no correlation was found between the observed expression changes (*rho* = -0.486, p = 0.329) (Fig 4).

On the bases of this observation, we evaluated whether IgG from the six tested NMOSD patients were directed to BMEC. Using Wb on BMEC lysate, we did not find any binding pattern neither with patients’ IgG (NMO_1-6_) nor Control-IgG (S2 Fig).

### 4. NMO-IgG induce alteration of BBB functional properties *in vivo* and *in vitro*

**Fig 5:**
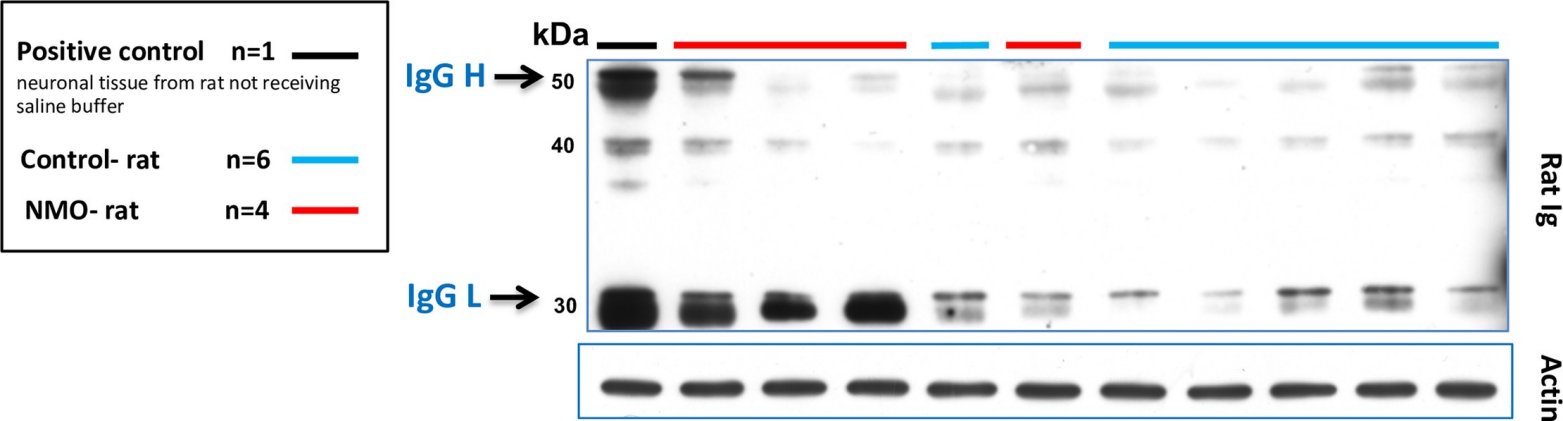
Rat IgG in periventricular areas of the CNS after seven days of NMO-IgG and Control-IgG infusions. The positive control was not perfused intracardially with saline perfusion. In this sample, both heavy (H) and light (L) immunoglobulin chains (black line) present in brain vessels could be identified. Three out of four saline perfused NMO-rats accumulated rat IgG (mainly L chain) in periventricular regions compared to Control-rats accumulating only traces of H and L chains. Actin was used as protein deposition control, same blot.

The observation, in *ex vivo* and *in vitro* experiments, that NMO-IgG induce structural modification on tight junction proteins, suggests that NMO-IgG may lead to an alteration of BBB functional properties. We evaluated the diffusion of rat-IgG in periventricular brain areas using immunoblotting on the brains of NMO-rats (n = 4) and Control-rats (n = 6). One extra rat (positive control) did not receive the saline intracardiac perfusion (thus with blood still trapped into the brain tissue), and was used to identify rat-IgG in Wb. Heavy (H) and light (L) rat-IgG chains were well detected in the brain of the positive control (blood still present in brain vessels). A higher proportion of NMO-rats (3 out of 4 rats) accumulated rat-IgG (mainly L chain) in the periventricular regions compared to Control-rat (only traces of H and L chains in 6 rats) (Fig 5).

These observations show that rat-IgG can cross the BBB, from the periphery to the CNS parenchyma, when the rat is chronically infused with NMO-IgG. This suggests that NMO-IgG present in the CSF compartment can modify BBB functional properties in the periventricular regions.

To further assess the effect of NMO-IgG on the BBB, we evaluated the functional properties of the BBB using a bicameral model simulating the blood-CNS barrier. First, we measured the blood-to-brain clearance rate of three NMO-IgG over 24 hours. NMO-IgG_2_ clearance rate (median 1.22 [1.12–1.75]) was twice that of the Control-IgG (median 0.54; [0.35–0.73]); p = 0.050). No other differences were found between the other NMO-IgG tested and the IgG-Control (Table 1). After exposing cells to the NMO-IgG or control-IgG, the permeability to sucrose was then measured over 3 hours. There was a significantly higher sucrose clearance rate in cells treated with NMO-IgG_1_ (median 0.42; [0.36–0.51]) and NMO-IgG_2_ (median 0.44; [0.41–0.48] than in those treated with Control-IgG (median 0.31; [0.29–0.34]; p = 0.050) (Table 1).These results indicate that NMO-IgG added in the blood compartment weaken the BBB.

**Table 1:** Permeability of an *in vitro* BBB model to immunoglobulins and sucrose.

Permeability, median (IQR)	Control-IgG	NMO-IgG_1_	NMOIgG_2_	NMO-IgG_3_	p-value*(Kruskal Wallis)*
IgG (ul.hr -1. cm-2)	0.54 (0.35–0.73)	0.98 (0.54–1.45)	1.22 (1.12–1.75)*	1.17 (0.30–1.18)	0.238
Sucrose (ul.min -1. cm-2)	0.31 (0.29–0.34)	0.42 (0.36–0.51)*	0.44 (0.41–0.48)*	0.39 (0.33–0.39)	**0.042**

*p = 0.050 *vs*. Control IgG

Abbreviation; IQR, interquartile range

BMEC were exposed to three different NMO-IgG (2 mg/ml) in the upper compartment of the culture system simulating blood. The basolateral side simulates the CSF. NMO-IgG_2_ clearance rate was twice that of the Control-IgG, p = 0.050. When exposing cells to the NMO-IgG or control-IgG, the sucrose clearance rate in cells treated with NMO-IgG_1_ and NMO-IgG_2_ was significantly higher than in those treated with Control-IgG, p = 0.050)

## Discussion

The present study evaluating the effect of purified IgG from AQP4-antibody positive NMOSD patients on BBB properties showed that: 1) NMO-IgG induce an inflammatory secretome profile in brain microvessels, 2) have a direct impact on the molecular structure of the BBB and 3) induce a moderate functional alteration of the BBB.

In line with previous studies, we found an upregulation of several cytokines and chemokines, notably IL-1RA, IL-1β, TNFα, CXCL10 and CXCL3. This inflammatory secretome has been found increased in the CSF or serum of NMOSD patients reflecting a cell recruitment to sites of inflammation or even BBB weakening [19–23]. Interestingly, we also found an up-regulation of IL-6, proposed to be a surrogate diagnostic and prognostic marker in NMOSD [19,24–26], enhancing plasmablast survival and AQP4-antibody secretion [27]. More recently, IL-6 receptor blockage treatments have been proposed as a promising therapeutic option for NMOSD [28,29]. Apart from its effect on plasmablasts, IL-6 could be involved in BBB alteration. Indeed, a recent study using astrocyte and endothelial cell co-cultures showed IL-6 production in astrocytes and AQP4 internalisation after exposure to NMO-IgG in the abluminal side of the culture (brain compartment) [5]. Authors also found a structural BBB impairment characterised by a decreased and discontinuous Claudin-5 immunoreactivity at tight junctions and a molecular leakage through the BBB that was reverted after IL-6 soluble receptor blocking [5]. Our data suggest that isolated capillaries may be a source of IL-6 production other than astrocytes, as previously described [20,30]. In summary, the specific inflammatory secretome induced by NMO-IgG in brain microvessels likely modulates a subsequent pathophysiological process characterised by the structural weakening and functional impairment of the BBB. Whether endothelial cells or pericytes remaining embedded in the basal membrane that delimit the isolated microvessels are the main source of these cytokines remains to be established.

In order to study the impact of NMO-IgG on BBB structure itself, we use an original *ex vivo* BBB mimicking the close relationship between NMO-IgG and brain microvessels. Fresh IBMs are known to maintain *in vivo* BBB properties, and have been previously used for the study of endothelial molecular transporters [31,32], drug pharmacokinetic or efflux transport regulation [33], and pathophysiology processes in animal models [34,35]. However, the application of this technique on NMOSD-related models has not been previously performed. The decrease in expression of Claudin-5 in IBMs when exposed to NMO-IgG in the absence of any previous contact with breaching substances (i.e, such as pertussis toxin used in animal models of cerebral autoimmune disease), suggests that NMO-IgG directly contribute to the structural destabilization of the BBB from the brain compartment. Moreover, we reproduced our findings seen in IBMs observing a similar decrease in Claudin-5 expression in BMECs treated with NMO-IgG. Two previous studies found a down-regulation of Claudin-5 in immortalised human brain microvascular endothelial cell lines treated with the serum of NMO patients but not in controls [7,8]. We underline that although there was an increase of Occludin expression in most of BMECs cultured with NMO-IgG, an increase higher than 20% was found only in two out of 6 BMEC. In addition, the effect of Control-IgG also resulted in an increase of up to 30% in Occludin expression, indicating that these changes may not be attributed specifically to NMO-IgG.

Whether AQP4 antibodies or other unknown auto-antibodies present in NMOSD patients are able to directly open the BBB is not completely resolved. Interestingly, we found that the decreased expression of Claudin-5 did not correlate with the loss of AQP4 in individual patients. This finding may suggest that other IgG, different from the specific AQP4-antibody, could be involved in BBB disruption as recently proposed [7–9]. In fact, GRP78, an antibody specific to the surface of endothelial proteins has been recently identified in the serum of patients with AQP4-antibodies and systemic lupus erythematous [9]. Another study described antibodies against BMEC in the serum of NMOSD patients that could alter BBB properties through an autocrine secretion of antivascular endothelial growth factor (VEGF) by the BMEC [7]. However, when performing Wb we did not observe any specific pattern of IgG binding to endothelial cells in the present study.

NMO-IgG may exert its pathological effect from the CSF compartment after passing through subpial spaces where vessels penetrate the brain parenchyma [4,11]. To validate this hypothesis *in vivo*, an intraventricular chronic infusion of NMO-IgG in rat brain was performed. This leads to a wide NMO-IgG distribution through CSF in the neural tissues [15]. By analysing brain microvessels from NMO-rats it was possible to detect not only the deleterious effect of NMO-IgG, concerning mainly the loss of Claudin-5 but also a functional alteration represented by the passage of rat-IgG from the blood to the CNS. AQP4-expressing astrocytic end-feet would be directly exposed to CSF NMO-IgG, thus triggering astrocyte damage and further BBB disruption [6].

## Conclusion

Overall, the present study provides a global perspective on the pathophysiological role of NMO-IgG at the BBB level. NMO-IgG induce a distinctive inflammatory secretome profile that may modulate the subsequent alterations of the BBB properties. The structural weakening is driven by the decrease of Claudin-5 expression both from the blood and the CSF compartments. The functional alteration is reflected by a higher permeability through brain microvessels allowing the passage of circulating molecules including IgG to the CNS when the antibody is present in the CSF compartment. To this regard, the NMO-IgG “by itself” may penetrate through the breaching barrier, distribute through the extracellular fluid and, finally, trigger the disease after binding to the AQP4 channel.

## Supporting information

S1 Raw images Raw western blots. (PDF)

S1 Fig Freshly isolated brain microvessel structure. (a) Fresh brain microvessels are depicted after isolation, (b) pericyte staining (PDGFR- β), (c) Claudin-5, (d) Occludin, (e) PECAM, and (f) GFAP.
(PDF)

S2 Fig Binding pattern of NMO-IgG_1-6_ and Control-IgG_1-2_ on a lysate of brain microvascular endothelial cells. *An extra NMO-IgG (NMO-IgG_7_) was used to perform this experiment.
(PDF)

## List of abbreviations

NMOSD: Neuromyelitis optica spectrum disorders
CNS: central nervous system
CSF: cerebrospinal fluid
BBB: blood brain barrier
IBMs: isolated brain microvessels
Wb: western blot
PBS: phosphate-buffered saline
IHC: immunohistochemical
AKRB: BSA-supplemented Kreps Ringer buffer
DMEM: Dulbecco's Modified Eagle's
ELISA: Enzyme-linked immunosorbent assays
DAPI: 4',6-diamidino-2-phenylindole
IQR: interquartile range
VEGF: antivascular endothelial growth factor
