# Supplementary material for: Purified IgG from aquaporin-4 neuromyelitis optica spectrum disorder patients alters blood-brain barrier permeability
Source: PLoS One. 2020 Sep 3;15(9):e0238301. doi: 10.1371/journal.pone.0238301 (PMC7470361; doi:10.1371/journal.pone.0238301)

RAW WESTERN BLOTS

-"X" is not shown in the original figure of the manuscript since some experiments such as isolated brain microvessels were performed in duplicate.

-Chemoluminescence detection for western blotting was performed with a commercial kit (Immobilon Western Chemiluminescent HRP Substrate WBKLS0500 Millipore Merck KGaA Darmstadt Germany.

-Band density was evaluated using Image J software.

Figure 3.a.

- **Claudin-5** and **actin** expression in cultures of isolated brain microvessels treated with NMO-IgG_1, Control-IgG and non-treated. The example is depicted in figure 3.a.

CLAUDIN AND ACTIN

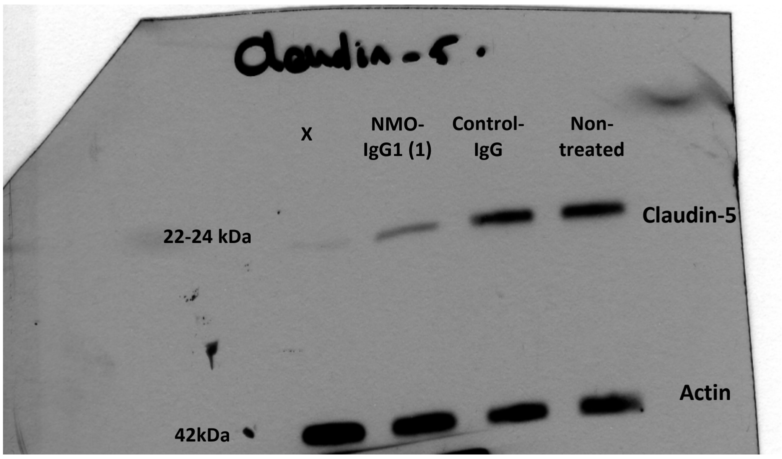

-**Occludin (up) and actin (down)** expression in cultures of isolated brain microvessels treated with NMO-IgG$_1$, Control-IgG and non-treated. The example is depicted in figure 3.a.

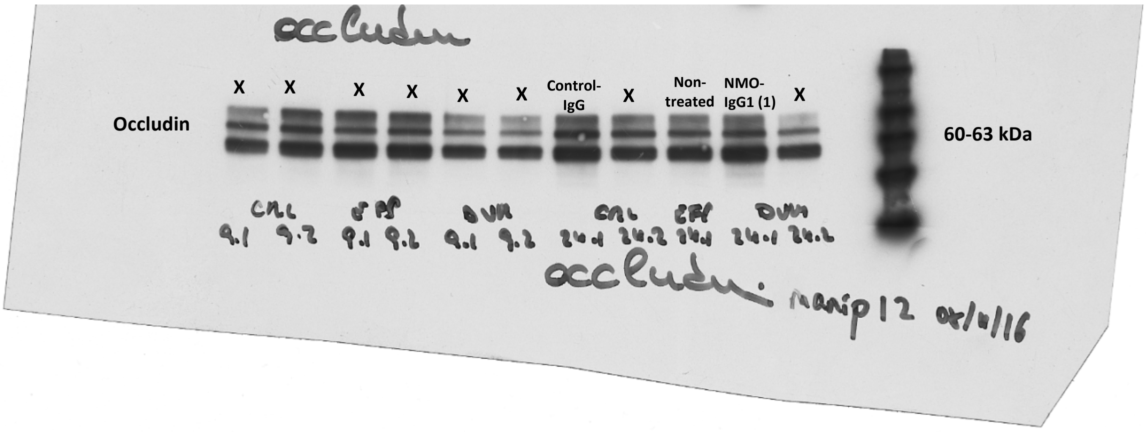

ACTIN

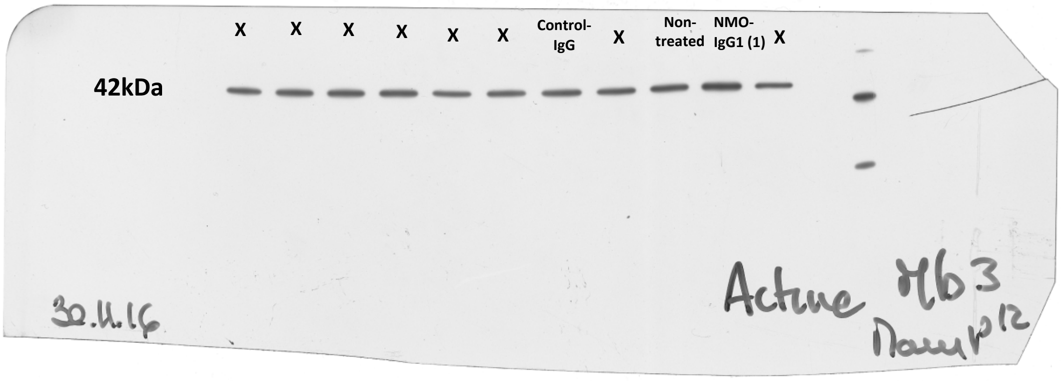

-Z0-1 (up) and actin (down) expression in cultures of isolated brain microvessels treated with NMO-IgG$_1$, Control-IgG and non-treated. The example is depicted in figure 3.a.

ZO-1

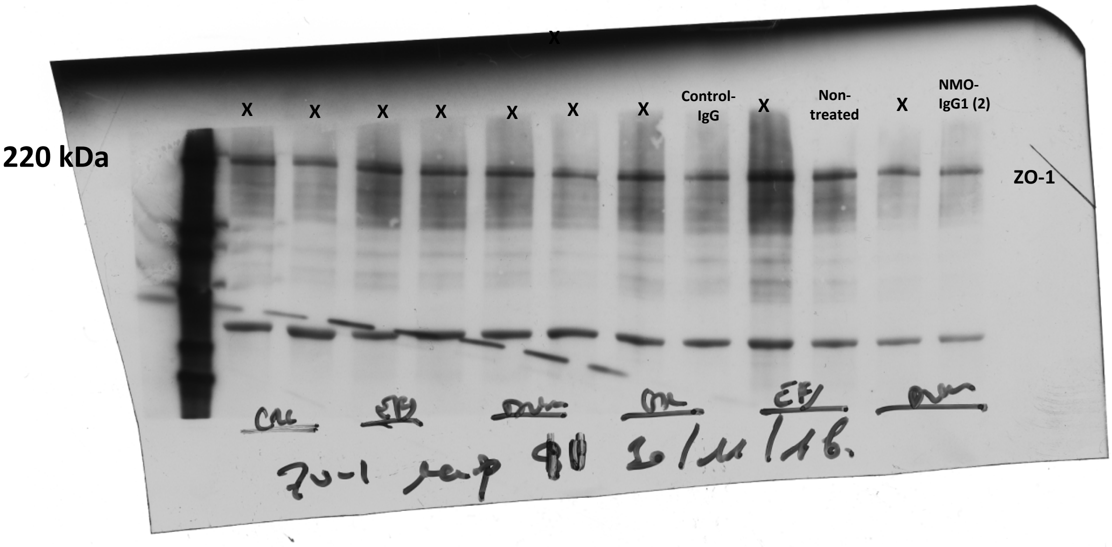

ACTINE

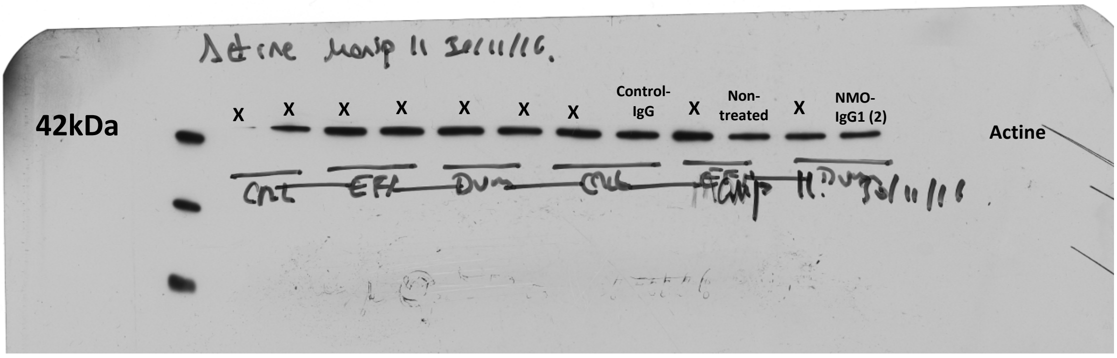

Figure 3.b

-**Actin** expression in cultures of endothelial cells treated with NMO-IgG_{1-6}, Control-IgG and non-treated. The example is depicted in figure 3.b.

ACTIN

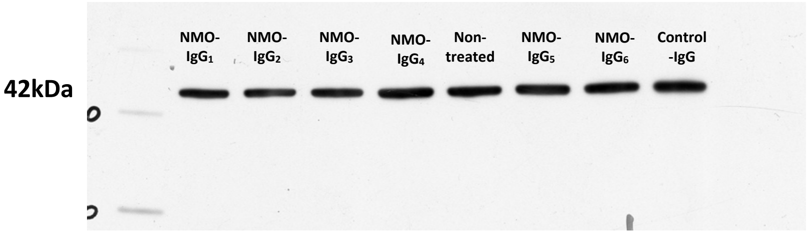

- **Claudin-5** expression in cultures of endothelial cells treated with NMO-IgG_{1-6}, Control-IgG and non-treated. The example is depicted in figure 3.b.

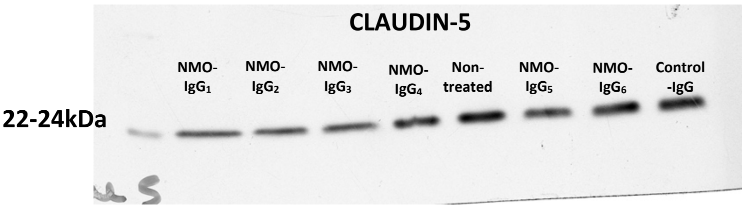

- **Occludin** expression in cultures of endothelial cells treated with NMO-IgG_{1-6}, Control-IgG and non-treated. The example is depicted in figure 3.b.

OCCLUDIN

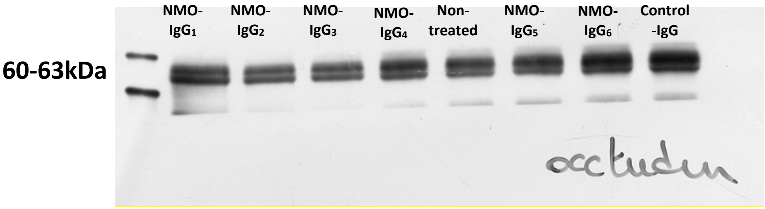

- **ZO-1** expression in cultures of endothelial cells treated with NMO-IgG_{1-6}, Control-IgG and non-treated. The example is depicted in figure 3.b.

ZO-1

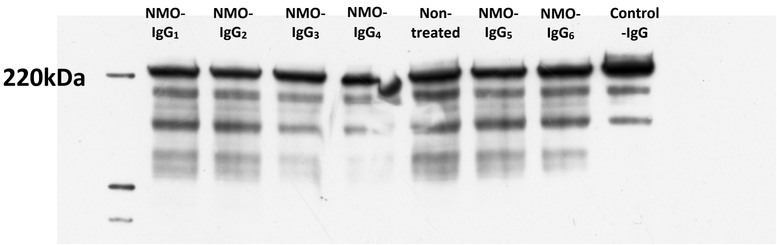

Figure 5.

-Rat IgG in periventricular regions from NMO-rat (label as 10,12,13,16) and Control-rat (14,17,18,21,25,26). The non-perfused rat (positive control) is labelled as 0. Heavy and light chains signals are located at 50 and 30 kDa of molecular weight

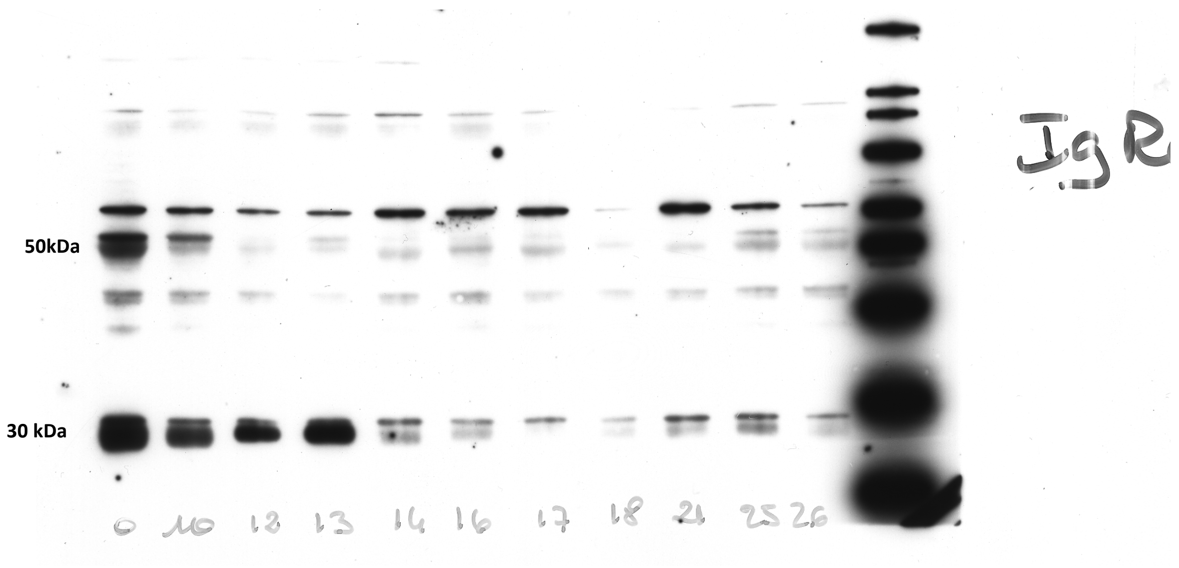

Actin corresponding to actin of figure 5 NMO-rat and Control-rat. Actin from NMO-rat (label as 10,12,13,16) and Control-rat (14,17,18,21,25,26). The non-perfused rat (positive control) is labelled as 0.

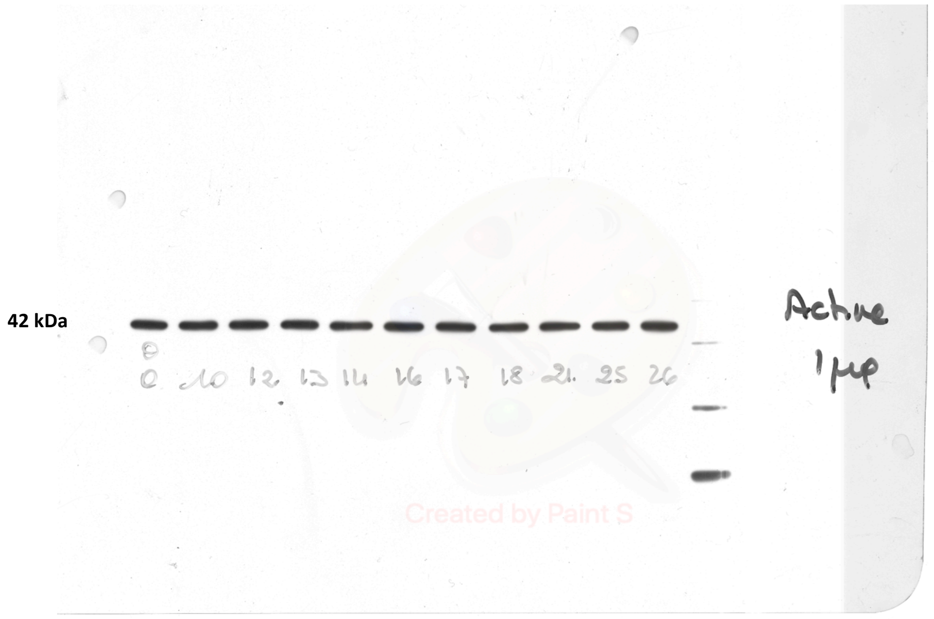

Supplement: S1 Raw images — (PDF) [file pone.0238301.s001.pdf]
