# Supplementary figures and images for: Purified IgG from aquaporin-4 neuromyelitis optica spectrum disorder patients alters blood-brain barrier permeability
Source: PLoS One. 2020 Sep 3;15(9):e0238301. doi: 10.1371/journal.pone.0238301 (PMC7470361; doi:10.1371/journal.pone.0238301)

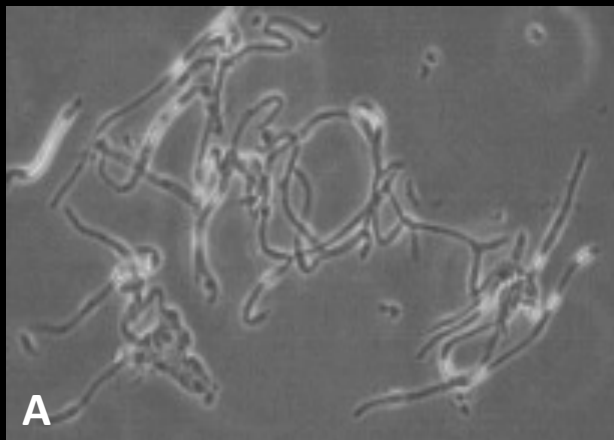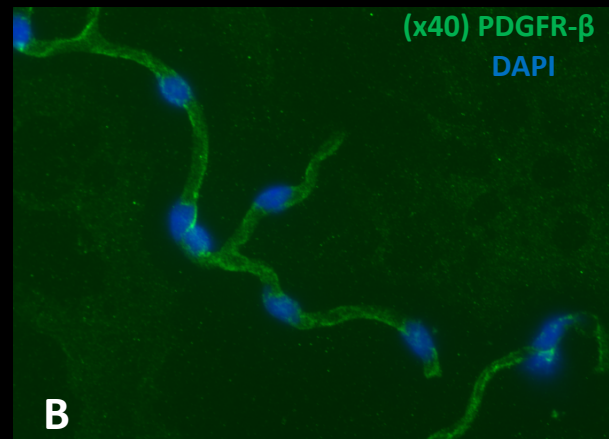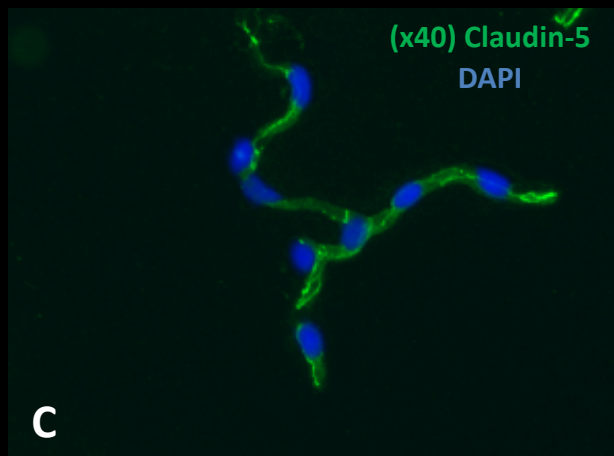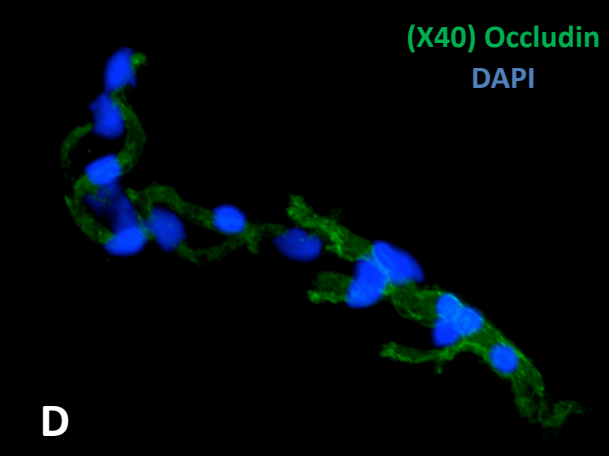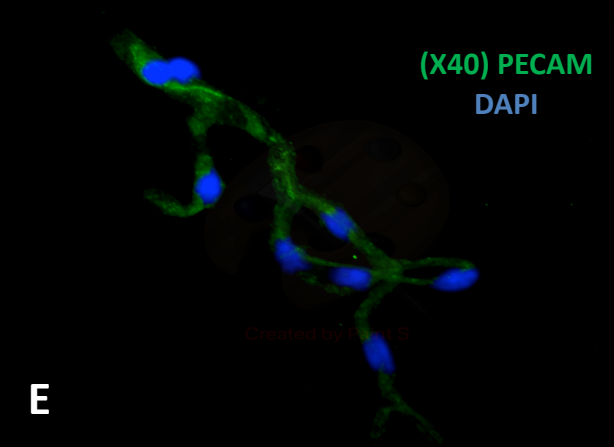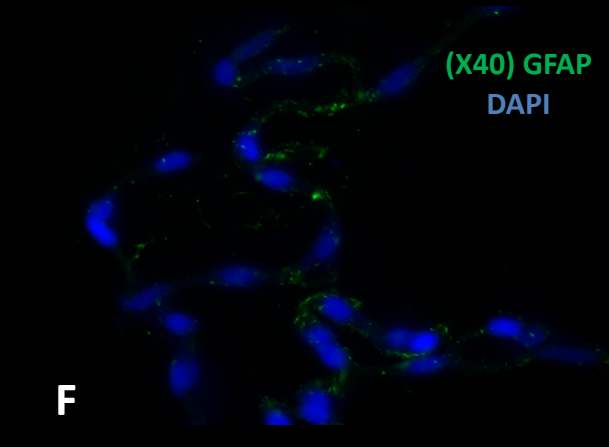

Supplement: S1 Fig — (a) Fresh brain microvessels are depicted after isolation, (b) pericyte staining (PDGFR- β), (c) Claudin-5, (d) Occludin, (e) PECAM, and (f) GFAP. (PDF) [file pone.0238301.s002.pdf]

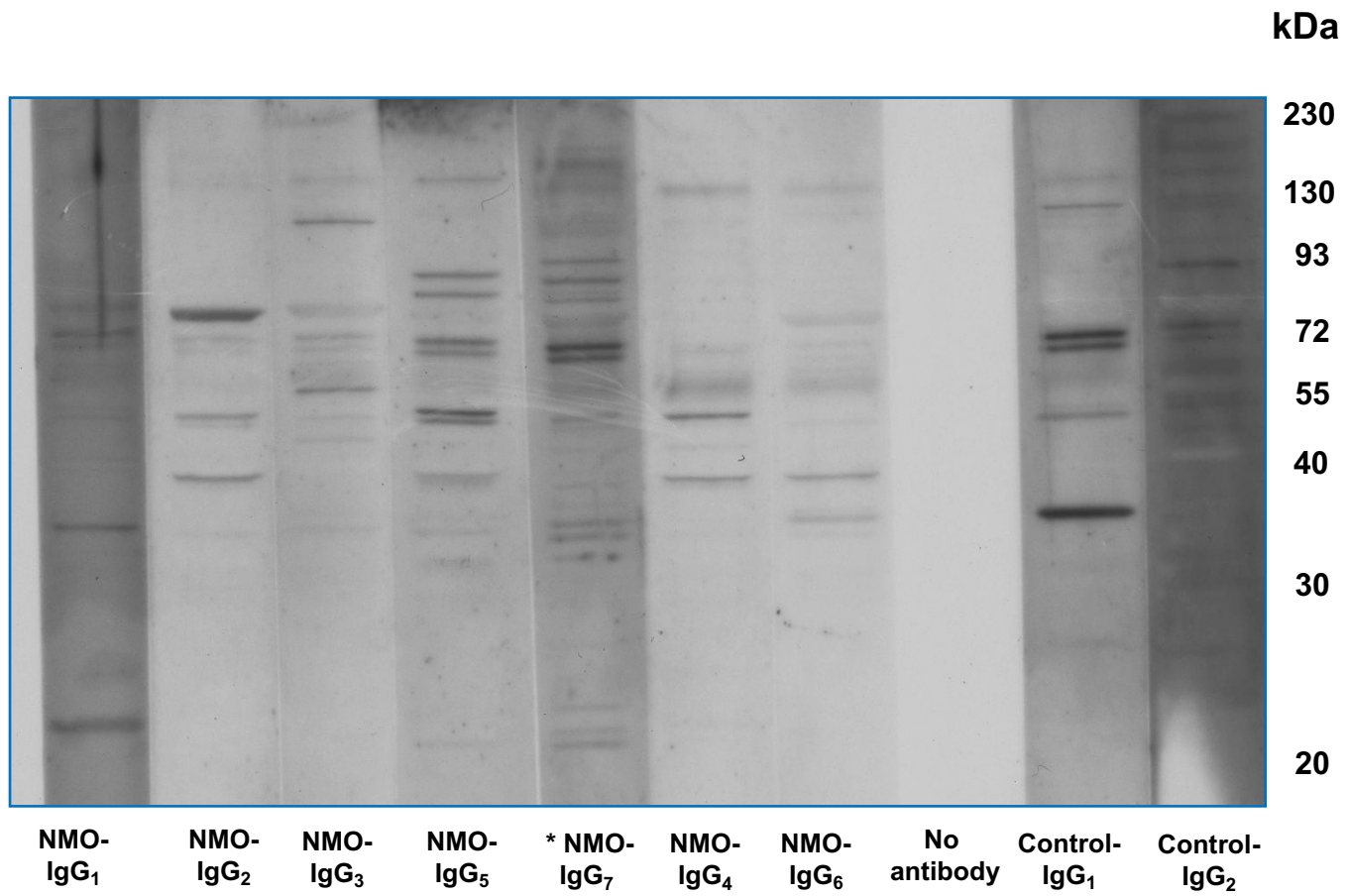

Supplement: S2 Fig — *An extra NMO-IgG (NMO-IgG7) was used to perform this experiment. (PDF) [file pone.0238301.s003.pdf]
